# A specialized CD107a^+^ macrophage subset drives hyperphagocytosis of mycobacteria

**DOI:** 10.1016/j.isci.2026.117622

**Published:** 2026-09-25

**Authors:** Carolina Eto, Gabriela Luiz, Hernandez M. Silva, Eduarda L. Munari, Daniel A.G.B. Mendes, Bianca K. Beck, Lucas Z. Mascarin, Marick Starick, Maria Cecília C. Canesso, Bernardo S. Reis, Guilherme Silveira, Juliano Bordignon, Yonne K.T. de Menezes, Daniel S. Mansur, Edroaldo Lummertz da Rocha, André Luiz Barbosa Báfica

**Affiliations:** 1 Department of Microbiology, Immunology and Parasitology, Universidade Federal de Santa Catarina, Florianópolis, Santa Catarina, Brazil; 2 Laboratory of Immunophysiology, Ragon Institute of Mass General, MIT, and Harvard, Cambridge, MA, USA; 3 Department of Biology, Massachusetts Institute of Technology, Cambridge, MA, USA; 4 Howard Hughes Medical Institute, Cambridge, MA, USA; 5 Laboratory of Mucosal Immunology, Rockefeller University, New York, NY, USA; 6 Instituto Carlos Chagas, ICC/Fiocruz, Curitiba, Paraná, Brazil

**Keywords:** macrophages, phagocytosis, CD107a/LAMP1, CD195/CCR5, infection

## Abstract

Macrophages are essential for pathogen clearance, yet phagocytic specialization among subsets is poorly defined. Bone marrow-derived macrophages cultured with L929 supernatant or macrophage colony-stimulating factor (M-CSF) separate into FSC^lo^SSC^lo^F4/80^lo^CD11b^lo^ (FSC^lo^SSC^lo^) and FSC^hi^SSC^hi^F4/80^hi^CD11b^hi^ (FSC^hi^SSC^hi^) subsets. Transcriptomic and functional analyses reveal that FSC^lo^SSC^lo^ cells possess a hyperphagocytic program driven by enhanced actin cytoskeleton regulators (e.g., Arp2/3) and pro-inflammatory signaling (NF-κB). These cells excel at internalizing *Mycobacterium tuberculosis* virulent H37Rv and BCG through actin-mediated, cytochalasin D-sensitive mechanisms. High surface CD107a (LAMP1) expression marks this hyperphagocytic subset and correlates strongly with mycobacterial uptake. FSC^lo^SSC^lo^ macrophages produce more TNF and IL-6 upon mycobacterial or Toll-like receptor 2 (TLR2)/TLR5 stimulation yet retain IFN-γ-mediated killing capacity. *In vivo*, CD107a^+^ alveolar macrophages in BCG-infected lungs preferentially capture bacilli and upregulate CD195, recapitulating the *in vitro* phenotype. These findings establish CD107a as a key surface marker of a hyperphagocytic macrophage subset, providing new mechanistic understanding relevant to tuberculosis host defense and therapeutic development.

## Highlights

• BM-derived FSC^lo^SSC^lo^F4/80^lo^CD11b^lo^ macrophage subset excels at mycobacteria uptake
• Hyperphagocytic macrophages mount rapid pro-inflammatory TNF/IL-6 responses
• CD107a identifies a pre-existing hyperphagocytic macrophage subset *in vitro* and *in vivo*
• BCG infection triggers CD195 upregulation selectively on CD107a^+^ alveolar macrophages

## Graphical abstract

**Figure:**
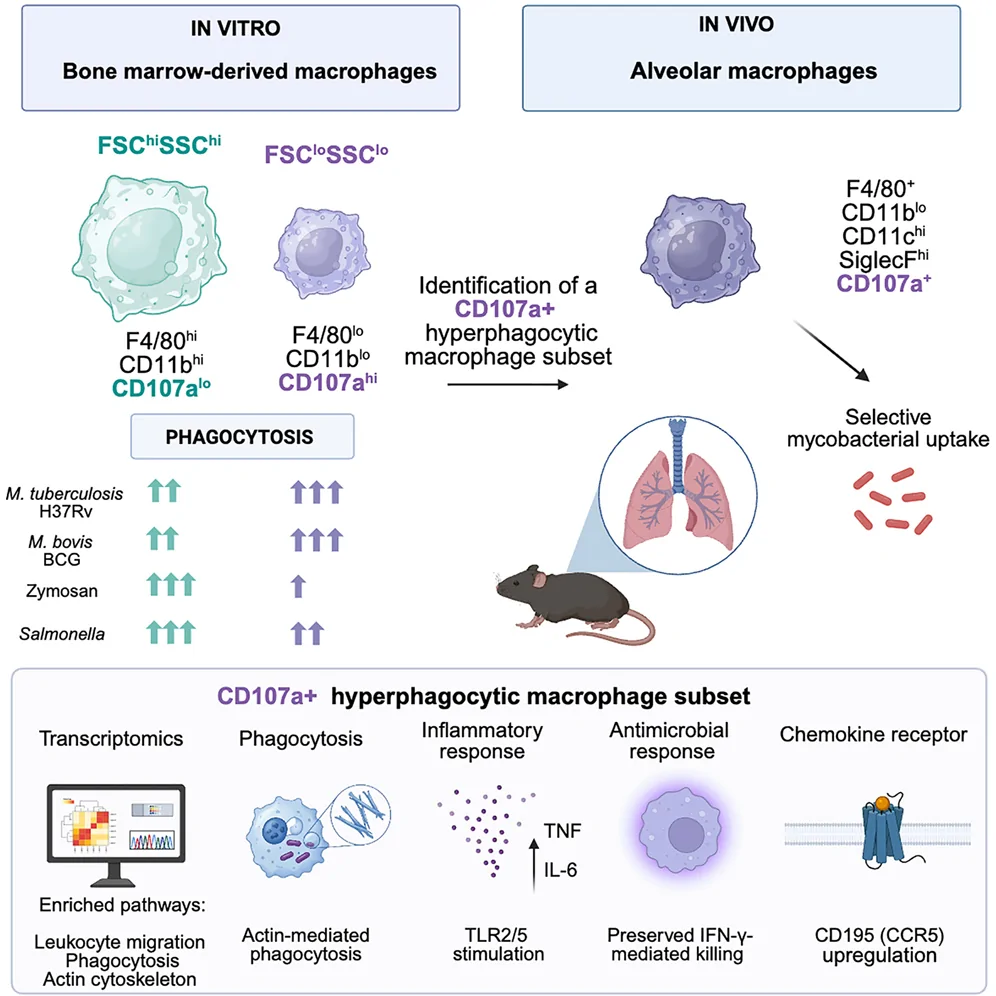

## Teaser

Immunology; Microbiology; Cell biology

## Introduction

Macrophages, critical immune cells orchestrating pathogen clearance, inflammation, and tissue homeostasis, exhibit remarkable diversity in ontogeny, phenotype, and function, a concept rooted in Metchnikoff’s foundational work on phagocytosis.^1^^,^^2^^,^^3^ Since the 1970s, bone marrow (BM)-derived macrophages (BMMs) cultured with L929-conditioned medium, a potent source of macrophage colony-stimulating factor (M-CSF), have been a cornerstone of macrophage research, enabling the generation of large, reproducible populations.^4^^,^^5^^,^^6^ Flow cytometry-based experiments have identified two distinct L929-generated BMM subsets: FSC^hi^SSC^hi^ macrophages, characterized by high F4/80 and CD11b expression, and FSC^lo^SSC^lo^ macrophages, marked by lower levels of these markers.^7^ However, the functional specialization of these populations, particularly their phagocytic and pathogen-specific roles, remains largely unexplored, presenting an opportunity to advance our understanding of macrophage heterogeneity.

Phagocytosis, a keystone of macrophage-mediated immunity, is a complex, multistep process, with pathogens like *Mycobacterium tuberculosis* exploiting these cells for survival and replication. During phagocytosis, macrophages recognize *M. tuberculosis* H37Rv through pattern recognition receptors, such as Dectin-1, Toll-like receptor 2 (TLR2), and mannose receptor (CD206), which bind bacterial cell wall components like lipoarabinomannan.^8^^,^^9^^,^^10^ This recognition triggers actin polymerization, driven by Arp2/3 complex and Rho GTPases, to engulf the bacteria into a phagosome, a process sensitive to inhibitors like cytochalasin D.^11^^,^^12^^,^^13^^,^^14^ Subsequently, phagosome maturation involves fusion with lysosomes, mediated by Rab GTPases, to form a phagolysosome where antimicrobial effectors attempt bacterial killing, though *M. tuberculosis* often evades destruction by inhibiting phagosome-lysosome fusion.^15^^,^^16^

In routine phagocytosis assays using L929-generated BMMs, we serendipitously observed that FSC^lo^SSC^lo^ macrophages exhibited superior uptake of *M. tuberculosis* virulent H37Rv and *M. bovis* BCG (Bacillus Calmette-Guérin) compared to FSC^hi^SSC^hi^ counterparts, challenging assumptions of functional uniformity by these cell cultures. This discovery prompted us to integrate transcriptomic profiling, phenotypic characterization, and functional assays comparing these subsets. Our results revealed that elevated surface expression of CD107a (LAMP1) and CD195 (CCR5) distinguishes hyperphagocytic macrophages with exceptional pathogen engulfment capacity. *In vivo*, these markers also identified a parallel subset among alveolar macrophages (AMs) in BCG-infected mice. This work uncovers a facet of macrophage biology, offering critical insights into host-mycobacteria interactions.

## Results

### Distinct actin and phagocytosis gene signatures define FSC^lo^SSC^lo^F4/80^lo^CD11b^lo^ and FSC^hi^SSC^hi^F4/80^hi^CD11b^hi^ macrophages

Building on prior studies,^7^ we identified two L929-generated BMM subsets by forward scatter (FSC) and side scatter (SSC) profiles (Figure 1A). Macrophages were categorized as FSC^lo^SSC^lo^ (∼20%, range 11%–45%), with low F4/80 and CD11b expression (Figures 1B–1D), and FSC^hi^SSC^hi^ cells (∼80%, range 51%–87%), with high surface F4/80 and CD11b expression (Figures 1B–1D). Similar subsets emerged in BM cell cultures treated with exogenous M-CSF, a key differentiation factor present in L929-conditioned media^5^ (Figures 1E–1H). Flow cytometry-based sorting (Figure 1I) and microscopy (Figure 1J) revealed smaller cell areas for FSC^lo^SSC^lo^ (∼19 μm^2^) than FSC^hi^SSC^hi^ macrophages (∼40 μm^2^) (Figure 1K), confirmed by FSC-based volume estimates (Figure 1L). Thus, differences observed in FSC/SSC (Figure 1A) reflect reduced FSC (cell size) and SSC (internal complexity/granularity) in the FSC^lo^SSC^lo^ subset. Strikingly, bulk mRNA sequencing of sorted subsets highlighted distinct transcriptional profiles. For instance, Gene Ontology (GO) analysis showed FSC^hi^SSC^hi^ macrophages to be uniquely enriched in pathways such as autophagy, reactive oxygen species metabolism, response to nutrient levels, complement activation, cell recognition, and lysosomal and vacuolar transport (Figure 1M). In contrast, FSC^lo^SSC^lo^ macrophages favored actin cytoskeleton organization, response to virus, cytokine signaling, and cellular extravasation (Figure 1M). Both subsets shared core macrophage functions such as antigen presentation, entry into host, phagocytosis, cell chemotaxis, and leukocyte migration (Figure 1M), but FSC^lo^SSC^lo^ macrophages exhibited greater enrichment in these processes. Unique sets of phagocytosis- and actin cytoskeleton-related genes distinguished the FSC^lo^SSC^lo^ and FSC^hi^SSC^hi^ subpopulations (Figure 1N). The FSC^lo^SSC^lo^ subset was enriched for specific regulators (e.g., Arp2/3 complex, *Pfn1*, and *Coro1a*) and receptors (e.g., *Mincle*) associated with mycobacterial uptake, consistent with its functional specialization for Mtb/BCG^17^^,^^18^^,^^19^ (Figure 1N, *Pfn1*: right image and *Coro1a*: left image). These results suggest specialized actin-mediated roles in phagocytic interactions by BMM subsets.

**Figure 1:**
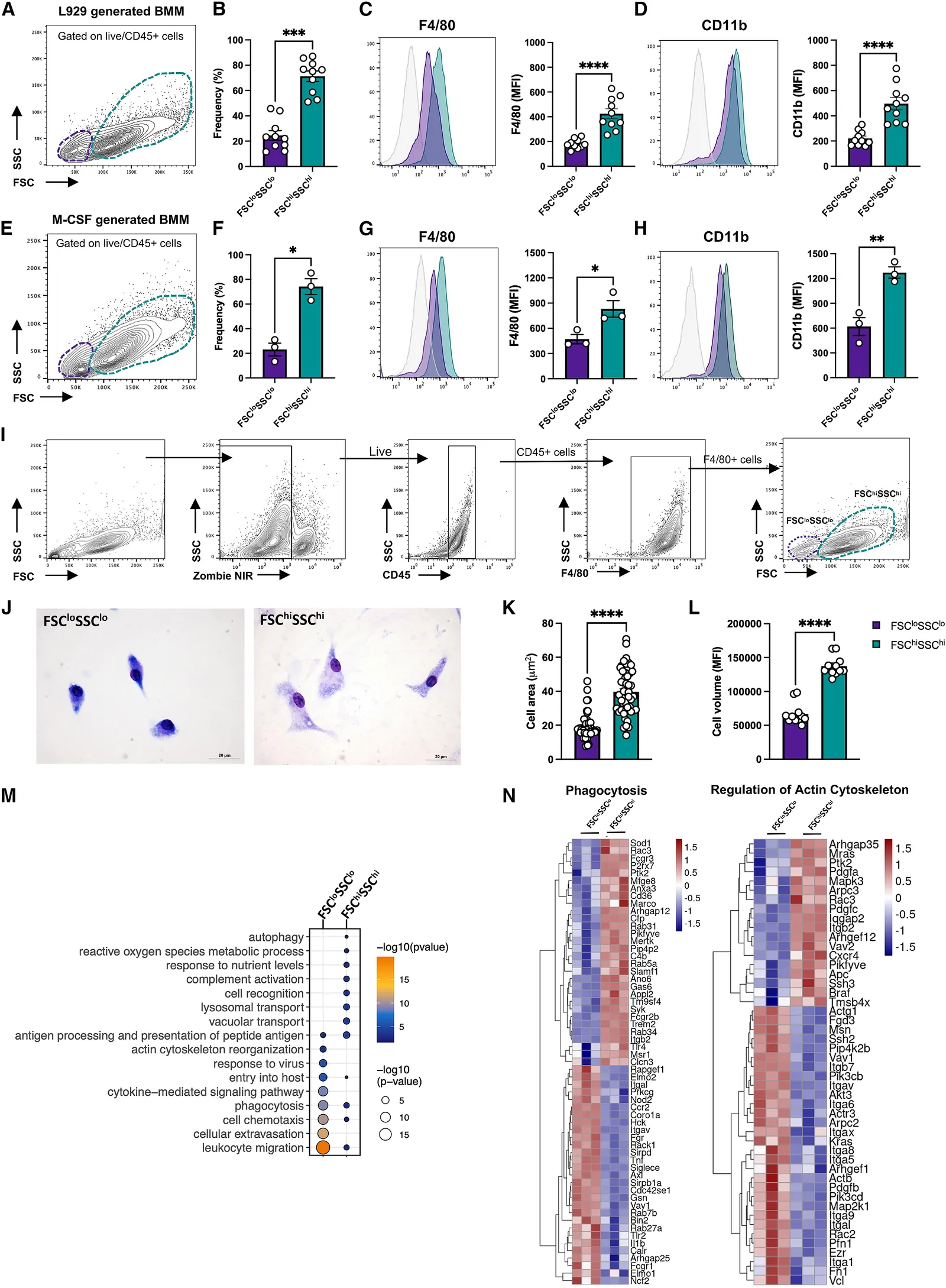
Distinct ‘actin/phagocytosis’ gene signatures in FSC^lo^SSC^lo^F4/80^lo^CD11^lo^ and FSC^hi^SSC^hi^F4/80^hi^CD11b^hi^ BM-derived macrophages (A) Bone marrow cells from C57BL/6J mice were cultured for 7 days in 20% L929-conditioned medium to generate bone marrow-derived macrophages (BMMs). Representative forward scatter (FSC) versus side scatter (SSC) dot plots of BMMs. (B) Frequencies of the two distinct BMM populations. (C) Representative overlaid histograms of F4/80 expression and MFI on live BMMs. (D) Representative overlaid histograms of CD11b expression and MFI on live BMMs. (E) Bone marrow cells from C57BL/6J mice were cultured for 7 days in M-CSF to generate BMMs. Representative FSC versus SSC dot plots of BMMs. (F) Frequencies of the two distinct BMM populations. (G) Representative overlaid histograms of F4/80 expression and MFI on live BMMs. (H) Representative overlaid histograms of CD11b expression and MFI on live BMMs. (I) Bone marrow cells from C57BL/6J mice were cultured for 7 days in 20% L929-conditioned medium, and BMMs were subsequently sorted by flow cytometry. Gating strategy for sorting F4/80^+^FSC^lo^SSC^lo^ and F4/80^+^FSC ^hi^SSC ^hi^ BMMs. (J) Representative panoptic-stained images of sorted FSC^lo^SSC^lo^ and FSC ^hi^SSC ^hi^ BMMs at 100× magnification; scale bar, 20 μm. (K) Quantitative analysis of cell area measured using ImageJ software. (L) Quantitative analysis of cell volume based on FSC MFI measured by flow cytometry. (M) Bubble plot depicting 16 enriched Gene Ontology (GO) terms among genes upregulated in steady-state sorted FSC^lo^SSC^lo^ and FSC ^hi^SSC ^hi^ BMMs. (N) Heatmaps showing the expression levels of differentially expressed genes in sorted FSC^lo^SSC^lo^and FSC ^hi^SSC ^hi^ BMMs, with rows representing individual genes and columns representing biological replicates. Data in (A–D) represent the mean ± SEM from 10 independent experiments performed in duplicates. Data in (E–H) represent the mean ± SEM from 3 independent experiments performed in duplicates. Data in (K) and (L) represent the mean ± SEM from 3 independent experiments, with 50 cells analyzed per experiment. Statistical analyses were performed using Student’s *t* test (∗*p* < 0.05; ∗∗*p* < 0.01; ∗∗∗*p* < 0.001; ∗∗∗∗*p* < 0.0001).

### Preferential mycobacterial phagocytosis by FSC^lo^SSC^lo^ macrophages

To investigate phagocytic interactions of BMM subsets, we performed *in vitro* assays using fluorescently labeled virulent H37Rv (Mtb-SYTO24) and BCG as a model system (Figure 2A). Binding assays, adapted from established protocols^9^ and quantified by flow cytometry, demonstrated that ∼75% of FSC^lo^SSC^lo^ macrophages associated with Mtb-SYTO24, compared to ∼40% of FSC^hi^SSC^hi^ macrophages, suggesting a higher binding affinity in the FSC^lo^SSC^lo^ subset (Figure 2B). This suggests that increased initial attachment facilitates subsequent actin-mediated phagocytosis in this population, consistent with the subset’s enrichment for specific actin regulators (e.g., Arp2/3 components) and receptors that support stable adhesion and zipper-like engulfment of mycobacteria. Consistent with this concept, phagocytosis assays further revealed that FSC^lo^SSC^lo^ BMMs associated with ∼70% of Mtb-SYTO24, significantly outperforming FSC^hi^SSC^hi^ macrophages, which internalized ∼30%, with elevated median fluorescence intensity (MFI) reflecting greater bacterial loads per FSC^lo^SSC^lo^ cell (Figure 2C). In contrast, FSC^hi^SSC^hi^ macrophages exhibited superior phagocytosis of zymosan-fluorescein isothiocyanate (FITC) (fungal derived) (Figure 2D) and *Salmonella*-SYTO24 (Gram-negative) (Figure 2E). Corroborating flow cytometry results, fluorescence microscopy of Mtb-SYTO24-infected macrophages showed that smaller cells (area < 30 μm^2^) contained an average of 2.52 bacilli per cell, compared to 1.32 bacilli in larger cells (> 30 μm^2^) (Figures 2F and 2G; *p* = 0.0011). Next, we compared uptake kinetics and capacity of FSC^lo^SSC^lo^ and FSC^hi^SSC^hi^ macrophages during exposure to *M. bovis* BCG. Time-course assays showed enhanced mycobacterial uptake by FSC^lo^SSC^lo^ macrophages from 1 h post-infection (Figures 2H and 2I). Moreover, quantitative four-parameter logistic regression of bacterial MOI (exposure) versus MFI values (uptake) confirmed greater infection burden in FSC^lo^SSC^lo^ macrophages compared to FSC^hi^SSC^hi^ cells across various MOIs (Figure 2J; R^2^ ∼0.99 for both subsets). Surprisingly, although FSC^hi^SSC^hi^ cells exhibited higher expression of classical mycobacterial phagocytosis receptors including CD206, Marco, CD36, Mcl, and Dectin-1 (Figures 1N and 3A), FSC^lo^SSC^lo^ macrophages demonstrated greater bacterial uptake.

**Figure 2:**
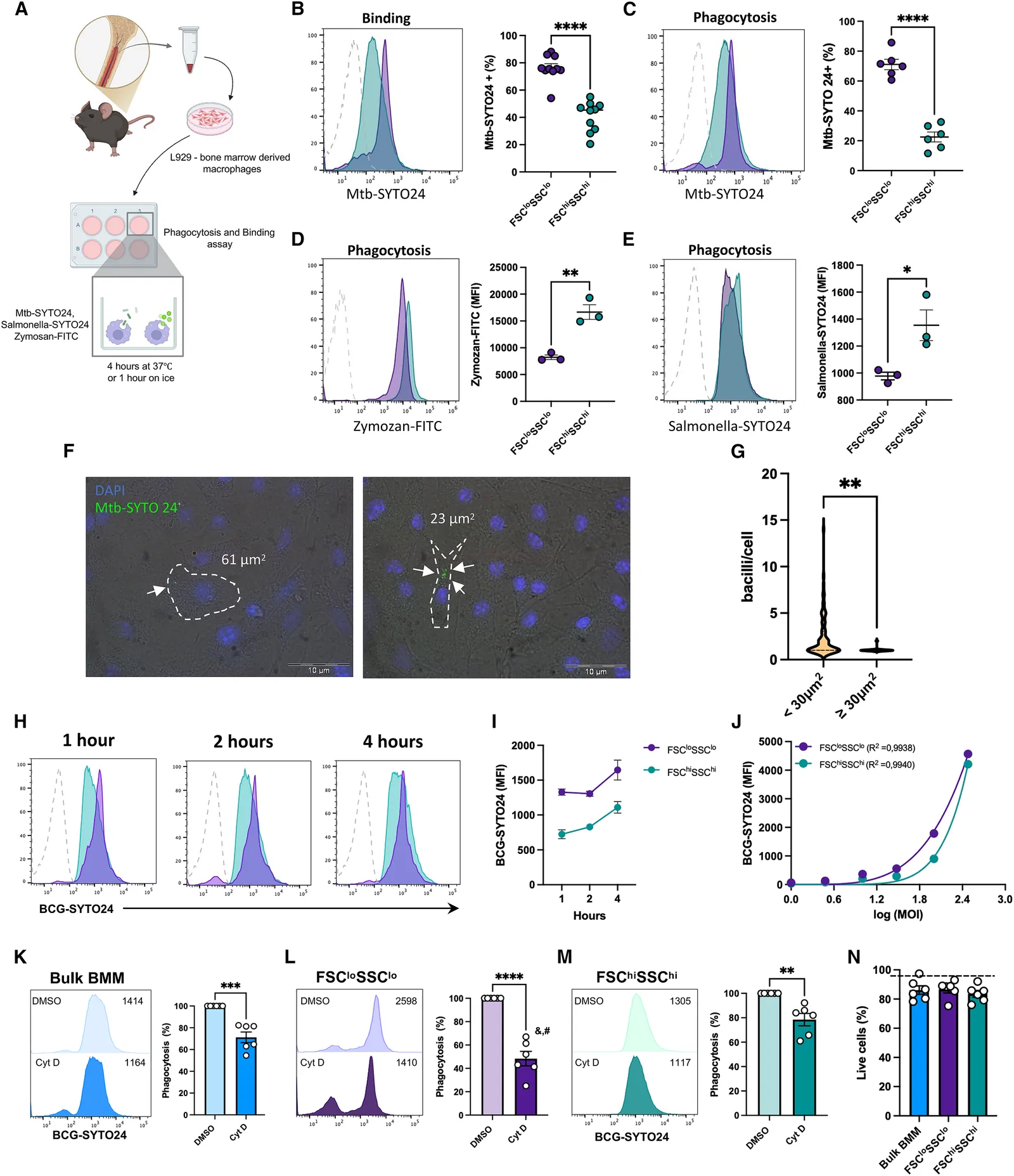
High phagocytic activity of BM-derived FSC^lo^SSC^lo^ macrophages toward mycobacteria (A) Experimental design. BMMs from C57BL/6J mice were exposed to *M*. *tuberculosis* H37Rv (Mtb)-SYTO24 (MOI 10) or zymosan-FITC (50 μg/mL) for 4 h at 37°C, or *Salmonella*-SYTO24 (MOI 10) for 30 min at 37°C, and particle association or phagocytosis was assessed by flow cytometry. (B) Representative histogram and frequency of Mtb-SYTO24^+^ FSC^lo^SSC^lo^ and FSC^hi^SSC^hi^ macrophages showing particle binding. (C) Representative histogram and frequency of SYTO24^+^ FSC^lo^SSC^lo^ and FSC^hi^SSC^hi^ macrophages showing phagocytosed Mtb. (D) Representative histogram and MFI of zymosan-FITC phagocytosis in FSC^lo^SSC^lo^ and FSC^hi^SSC^hi^ macrophages. (E) Representative histogram and MFI of *Salmonella*-SYTO24 phagocytosis in FSC^lo^SSC^lo^ and FSC^hi^SSC^hi^ macrophages. (F) BMMs were exposed to Mtb-SYTO24 (MOI 10) for 4 h at 37°C and analyzed by fluorescence microscopy. Representative images of two macrophages showing cell areas and fluorescent mycobacteria; nuclei were labeled with DAPI and Mtb with SYTO24. Scale bar, 10 μm. (G) Violin plot showing the number of bacilli per macrophage in 96 Mtb-SYTO24-infected macrophages, quantified from 50 fields across 2 independent experiments. (H) BMMs were exposed to *M*. *bovis* BCG-SYTO24 (MOI 10) for the indicated time points, and phagocytosis was assessed by flow cytometry. Representative histograms of BCG-SYTO24 uptake in FSC^lo^SSC^lo^ and FSC^hi^SSC^hi^ macrophages. (I) MFI of BCG-SYTO24 phagocytosis over time in FSC^lo^SSC^lo^ and FSC^hi^SSC^hi^ macrophages. (J) Sigmoid curve fit of BCG-SYTO24 multiplicity of infection (MOI; exposure) versus MFI (uptake) based on 6 independent experiments, as described in the Methods. (K) BMMs were pretreated with cytochalasin D (5 μg/mL) for 30 min, followed by exposure to BCG-SYTO24 (MOI 10) for 4 h at 37°C, and analyzed by flow cytometry. MFI of BCG-SYTO24 phagocytosis in bulk BMMs. (L) MFI of BCG-SYTO24 phagocytosis in FSC^lo^SSC^lo^ macrophages. (M) MFI of BCG-SYTO24 phagocytosis in FSC^hi^SSC^hi^ macrophages. (N) Frequency of live bulk BMMs, FSC^lo^SSC^lo^, and FSC^hi^SSC^hi^ macrophages following cytochalasin D treatment. Data represent the mean ± SEM from 10 (B), 6 (C) and 3 (D) and (E) independent experiments performed in duplicates. Data in (H) and (I) represent the mean ± SEM from 2–4 independent experiments. Data in (K–N) represent the mean ± SEM from 3 independent experiments. Statistical analyses were performed using Student’s *t* test. (∗*p* < 0.05; ∗∗*p* < 0.01; ∗∗∗*p* < 0.001; ∗∗∗∗*p* < 0.0001) or one-way ANOVA (#*p* < 0.05; &*p* < 0.01 for Cytochalasin D-treated bulk BMMs vs. FSC^lo^SSC^lo^ and FSC^hi^SSC^hi^ vs. FSC^lo^SSC^lo^ macrophages, respectively).

**Figure 3:**
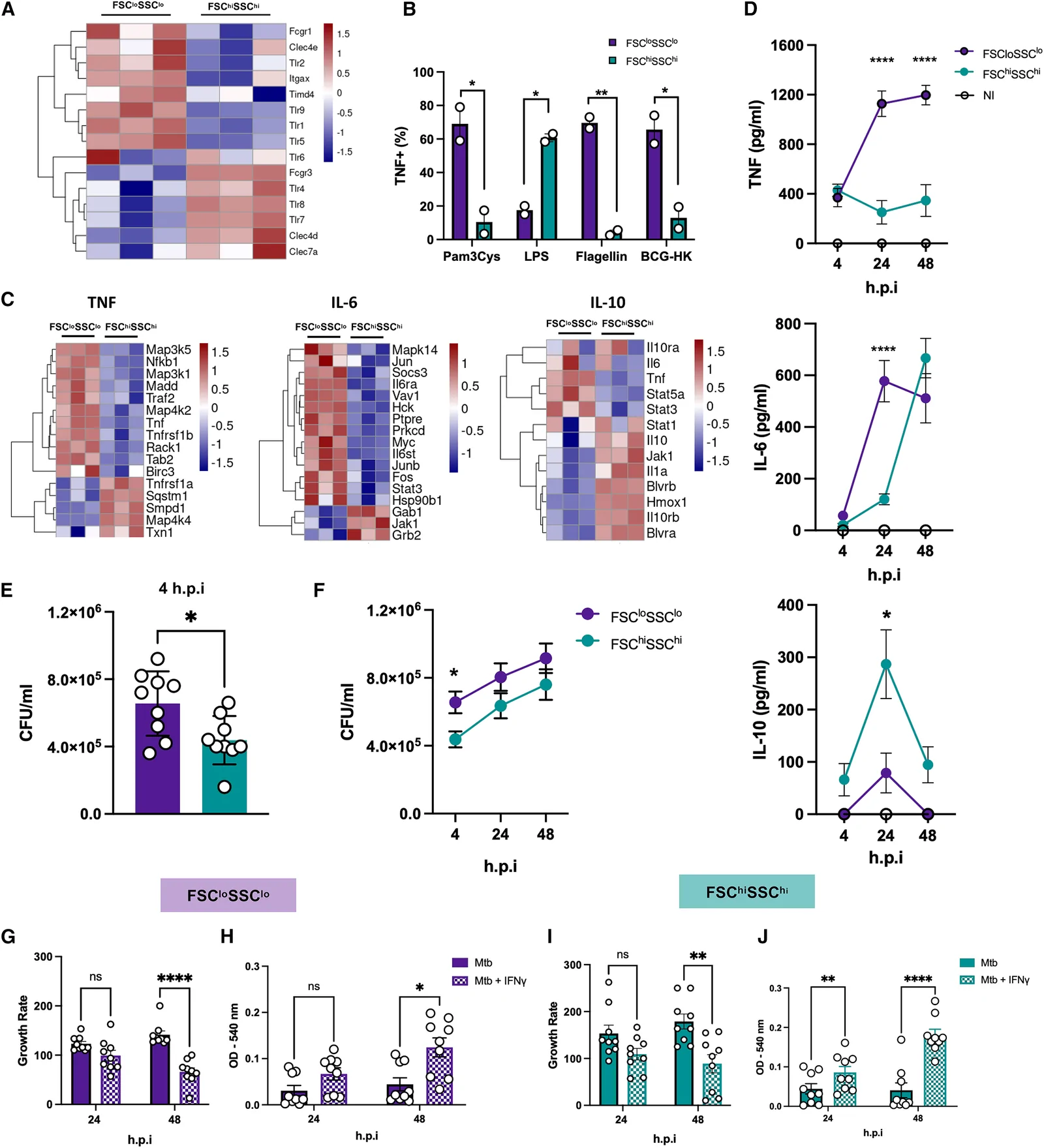
Differential TLR recognition and cytokine production in BM-derived FSC^lo^SSC^lo^ macrophages (A) Heatmap of differentially expressed innate immune receptor genes in sorted FSC^lo^SSC^lo^ and FSC^hi^SSC^hi^ BMMs from C57BL/6J mice (as in Figure 1I). (B) Bulk BMMs were stimulated with Pam3CSK4 (100 ng/mL), *E*. *coli* LPS (100 ng/mL), or flagellin (100 ng/mL) for 2 h, or heat-killed BCG (MOI 10) for 4 h at 37°C. Intracellular TNF was quantified by flow cytometry. Percentage of TNF-producing FSC^lo^SSC^lo^ and FSC^hi^SSC^hi^ macrophages following stimulation with the indicated microbial stimuli. (C) Heatmaps depicting expression levels of differentially expressed genes associated with TNF, IL-6, and IL-10 signaling pathways in sorted FSC^lo^SSC^lo^ and FSC^hi^SSC^hi^ BMMs. (D) Cytokine production by purified FSC^lo^SSC^lo^F4/80^lo^CD11b^lo^ and FSC^hi^SSC^hi^F4/80^hi^CD11b^hi^ macrophages following Mtb exposure at 4, 24 and 48 h. (E) Phagocytosis assessed by colony forming unit (CFU) counts. (F) Intracellular bacterial growth assessed by CFU counts. (G) Mycobacterial growth rates at 24 h and 48 h, calculated as ratios relative to the 4-h time point in FSC^lo^SSC^lo^ macrophages exposed to Mtb with or without IFN-γ (100 IU/mL). (I) Mycobacterial growth rates at 24 h and 48 h, calculated as ratios relative to the 4-h time point in FSC^hi^SSC^hi^ macrophages exposed to Mtb with or without IFN-γ (100 IU/mL). (H, J) Nitrite levels in culture supernatants from the macrophages shown in (G) and (I), respectively. Data in (A) represent the mean ± SEM from 3 (A) and 2 (B) independent experiments. Data in (C), (D–J) represent the mean ± SEM from 3 independent experiments. Statistical analyses were conducted using Student’s *t* test (E and B) or two-way ANOVA with Tukey’s multiple comparisons test (D and F–J). ∗*p* < 0.05; ∗∗*p* < 0.01; ∗∗∗*p* < 0.001; ∗∗∗∗*p* < 0.0001.

Next, we investigated the phagocytic mechanisms of FSC^lo^SSC^lo^ and FSC^hi^SSC^hi^ BMMs, which showed distinct gene expression profiles for phagocytosis and actin cytoskeleton regulation (Figure 1N). We tested whether actin-mediated processes underpin their differential uptake of mycobacteria by treating cells with cytochalasin D, an actin polymerization inhibitor, and quantifying phagocytosis via flow cytometry, as established previously.^12^ In bulk BMMs, cytochalasin D reduced mycobacterial uptake by 28% (range: 18%–46%, *p* < 0.001, treated vs. vehicle; Figure 2K). Notably, FSC^lo^SSC^lo^ macrophages exhibited greater sensitivity, with a 49% reduction in phagocytosis (range: 33%–75%, *p* < 0.0001, treated vs. vehicle; Figure 2L) compared to a 21.7% reduction in FSC^hi^SSC^hi^ macrophages (range: 8%–39%, *p* < 0.01, treated vs. vehicle; Figure 2M). Although FSC^lo^SSC^lo^ macrophages exhibited superior phagocytosis of mycobacteria (Figures 2B, 2C, 2H, 2I, and S1A), this phenotype was accompanied by a broader increase in actin dependence. Parallel dose-response experiments demonstrated that the FSC^lo^SSC^lo^ subset also displayed greater sensitivity to cytochalasin D during internalization of *Salmonella* and zymosan particles compared with the FSC^hi^SSC^hi^ subset (Figures S1A and S1B), without loss of cell viability (Figure 2N). These results suggest that the increased reliance on cytochalasin D-sensitive, actin-mediated mechanisms in FSC^lo^SSC^lo^ macrophages reflects a general feature of their phagocytic program rather than a pathogen-specific trait.

### Differential TLR sensing and cytokine responses by FSC^lo^SSC^lo^F4/80^lo^CD11b^lo^ macrophages

Transcriptomics revealed enriched innate sensing genes (e.g., *Tlr1*, *Tlr2*, and *Tlr5*) in FSC^lo^SSC^lo^ macrophages, while FSC^hi^SSC^hi^ cells favored *Clec7a* (Dectin-1) and *Tlr4* (Figure 3A). These transcriptomic findings were further validated by exposing bulk BMMs to known TLR agonists *in vitro* and measuring intracellular TNF accumulation via flow cytometry (Figure 3B). TLR agonist stimulation confirmed FSC^lo^SSC^lo^ macrophages as primary TNF producers for Pam3Cys, HK-BCG, and Flagellin, whereas ultra-pure *E. coli* lipopolysaccharide (LPS) triggered TNF in FSC^hi^SSC^hi^ cells (Figure 3B). Enhanced *Clec7a* expression aligned with increased phagocytosis of zymosan, a Dectin-1 ligand,^20^ by FSC^hi^SSC^hi^ macrophages (Figure 2D). FSC^lo^SSC^lo^ macrophages showed pro-inflammatory gene enrichment (TNF and IL-6), while FSC^hi^SSC^hi^ cells leaned toward IL-10 signaling (Figure 3C). Next, we used flow cytometry-based cell sorting to purify FSC^hi^SSC^hi^ and FSC^lo^SSC^lo^ macrophages and investigated the kinects of cytokine secretion upon mycobacteria stimulation. Culture supernatants from sorted FSC^lo^SSC^lo^ macrophages at 24 and 48 h post-Mtb exposure showed significantly higher TNF levels and lower IL-10 levels compared to FSC^hi^SSC^hi^ macrophages (Figure 3D). For IL-6 production, FSC^lo^SSC^lo^ macrophages exhibited elevated levels at 24 h post-infection, whereas FSC^hi^SSC^hi^ macrophages peaked at 48 h (Figure 3D). Despite higher Mtb uptake by FSC^lo^SSC^lo^ cells (Figure 3E), both subsets exhibited similar intracellular pathogen growth (Figure 3F) and IFN-γ-induced killing at 24 and 48 h (Figures 3G and 3I), with comparable nitrite production (Figures 3H and 3J). Collectively, these findings indicate that FSC^lo^SSC^lo^ macrophages strongly respond to TLR2 and TLR5 ligands and maintain IFN-γ-mediated mycobacterial killing.

### Surface CD107a and CD195 in FSC^lo^SSC^lo^ macrophages mark enhanced mycobacterial uptake *in vitro*

To further characterize macrophage subset phenotypes, we conducted flow cytometry using a comprehensive panel of surface markers, aiming to identify markers associated with enhanced mycobacterial uptake in L929-induced BMMs. FSC^hi^SSC^hi^ macrophages expressed higher levels of CD45, CD64, CD74, CD88, CD115, CD206, and CD207, while FSC^lo^SSC^lo^ macrophages showed elevated CD107a (LAMP1) and CD195 (CCR5) (Figures 4A and 4B), consistent across M-CSF- and L929-derived BMMs. Next, we assessed marker dynamics during BCG infection. While CD107a expression remained unchanged in both subsets, CD195 increased ∼5-fold in FSC^lo^SSC^lo^ macrophages versus ∼2-fold in FSC^hi^SSC^hi^ cells (Figures 4C and 4D) following BCG exposure. Notably, in infected FSC^lo^SSC^lo^ macrophages, the CD107a^+^ subset exhibited even higher mycobacterial loads compared to their CD107a^−^ counterparts (Figure 4E). Surface CD107a levels remained high and stable post-phagocytosis in time-course analyses (data not shown), suggesting its role as a marker rather than an uptake mediator. Furthermore, flow cytometry analysis of infected macrophages from 6 independent experiments revealed a strong positive monotonic correlation between BCG and CD107a MFI values (Spearman’s ρ ∼1.0, *p* = 0.0028) and a moderate correlation between BCG and CD195 MFI values (Spearman’s ρ ∼0.83, *p* = 0.058) in FSC^lo^SSC^lo^ macrophages (Figure 4F). Given its reported role in binding mycobacterial molecules in human dendritic cells,^21^ we investigated the potential involvement of CD195 in mycobacterial uptake by FSC^lo^SSC^lo^ macrophages using BMMs from CD195-deficient mice. Flow cytometry after 4 h Mtb exposure showed that CD195-deficient FSC^lo^SSC^lo^ macrophages retained bacterial phagocytosis (Figures 4G–4I). Although CD206 expression was elevated in FSC^hi^SSC^hi^ macrophages (Figure 4A), its lower expression in FSC^lo^SSC^lo^ cells suggests that this mannose receptor is unlikely to contribute to their selective mycobacterial binding/uptake, pointing to alternative receptors or mechanisms. Nevertheless, our *in vitro* data suggest CD107a and CD195 may serve as surface markers for identifying macrophages with heightened phagocytic activity toward mycobacteria.

**Figure 4:**
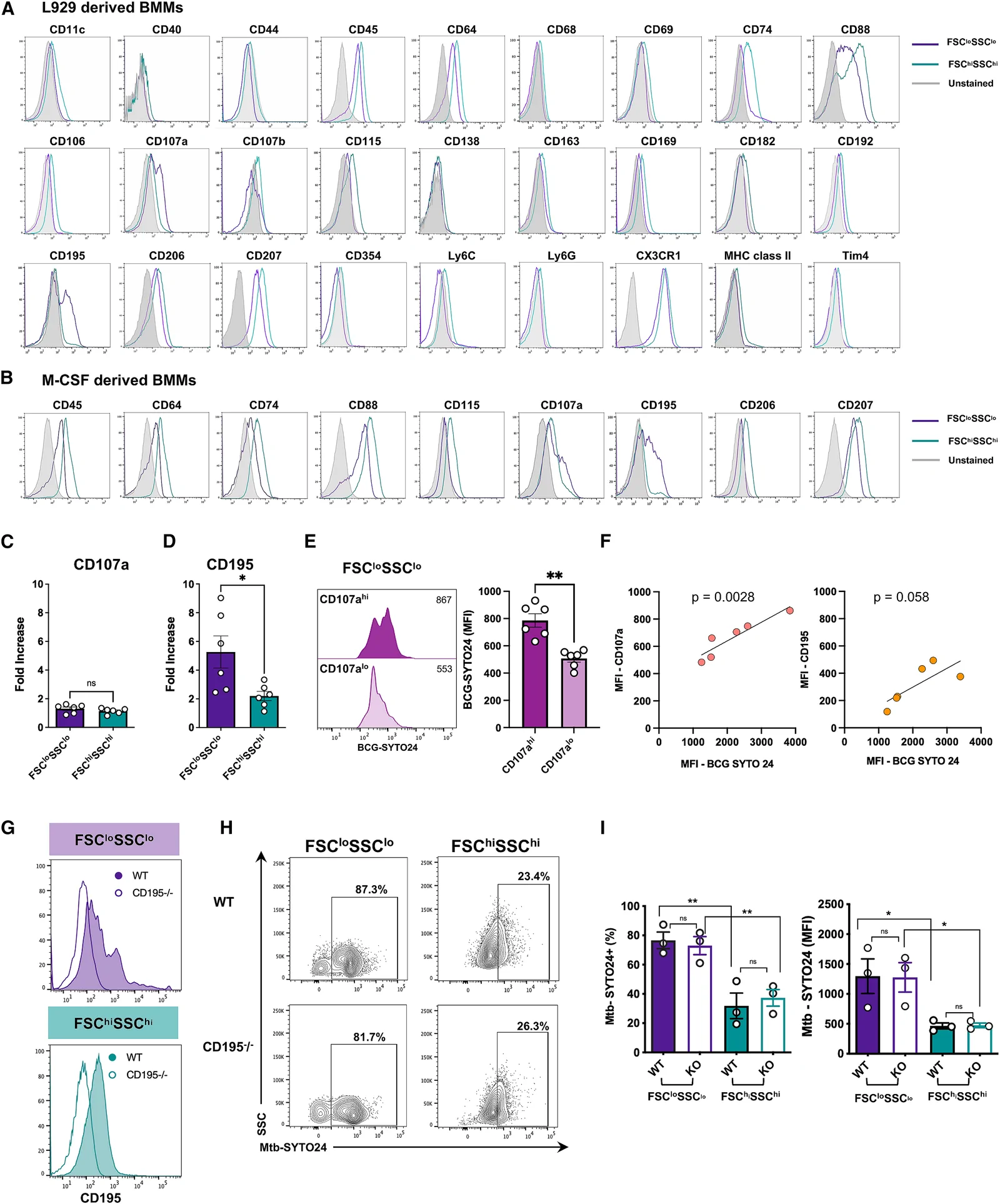
Surface expression of CD107a and CD195 in FSC^lo^SSC^lo^ and FSC^hi^SSC^hi^ macrophage subsets (A) Representative histograms of surface marker expression in FSC^lo^SSC^lo^ and FSC^hi^SSC^hi^ BMMs generated with L929-conditioned medium. (B) Representative histograms of surface marker expression in FSC^lo^SSC^lo^ and FSC^hi^SSC^hi^ BMMs generated with M-CSF. (C) BMMs were exposed to BCG (MOI 10) for 4 h, and surface expression was assessed by flow cytometry. Fold increase of MFI of surface CD107a in FSC^lo^SSC^lo^ and FSC^hi^SSC^hi^ BMMs relative to uninfected cells. (D) Fold increase of MFI of surface CD195 in FSC^lo^SSC^lo^ and FSC^hi^SSC^hi^ BMMs relative to uninfected cells. (E) Comparison of BCG-SYTO24 MFI between CD107a^hi^ and CD107a^lo/-^ in infected FSC^lo^SSC^lo^ macrophages gated on BCG^+^ events. (F) Scatter plots showing correlations between BCG MFI and surface marker expression in FSC^lo^SSC^lo^ macrophages at 4 h post-BCG exposure from 6 independent experiments. Left, correlation between BCG and CD107a MFI (Spearman’s ρ = 1.0, *p* = 0.0028, *n* = 6). Right, correlation between BCG and CD195 MFI (Spearman’s ρ ≈ 0.83, *p* = 0.058, *n* = 6). (G) BMMs from C57BL/6J and CD195^−/−^ mice were exposed to Mtb-SYTO24 (MOI 10) for 4 h and analyzed by flow cytometry. Representative histograms confirming CD195 deficiency in in CD195^−/−^ BMMs. (H) Representative dot plots showing the frequency of Mtb-SYTO24^+^FSC^lo^SSC^lo^ and FSC^hi^SSC^hi^ macrophages in WT and in CD195^−/−^ BMMs. (I) Frequency and MFI of Mtb-SYTO24-exposed WT and CD195^−/−^ FSC^lo^SSC^lo^ and FSC^hi^SSC^hi^ macrophages. Data in (A) and (B) represent the mean ± SEM from 3 independent experiments. Data in (C–E) and (G–I) represent the mean ± SEM from 3 independent experiments. Data in (F) represent 6 independent experiments and circles represent MFI values plotted on linear axes. Statistical analyses were performed using Student’s *t* test for panels C–E and I. Correlations in panel (F) were assessed using Spearman’s rank correlation. ∗*p* < 0.05; ∗∗*p* < 0.01.

### CD107a identifies hyperphagocytic AMs *in vivo*

To investigate whether these surface molecules serve as markers for hyperphagocytic activity by macrophages *in vivo*, we examined lung macrophages in C57BL/6J mice using an aerogenic BCG infection model.^22^ Lung macrophages were categorized as AMs (Live/CD45^+^CD64^+^F4/80^+^CD11b^lo^SiglecF^hi^CD11c^hi^) or interstitial macrophages (IMs, Live/CD45^+^CD64^+^F4/80^+^CD11b^hi^SiglecF^−^CD11c^lo^) based on established flow cytometry panels (Figure 5A).^23^^,^^24^^,^^25^^,^^26^ Consistent with prior findings,^22^ AMs displayed significantly greater association with fluorescent BCG bacilli compared to IMs at 18 h post-infection, as assessed by flow cytometry (Figures 5B and 5C). We then quantified surface expression of CD107a and CD195 on AMs and IMs from saline- and BCG-exposed mice. AMs exhibited higher baseline expression of both markers compared to IMs (Figures 5D and 5E). Post-infection, CD107a levels remained unaltered in AMs, aligning with *in vitro* observations (Figure 4C), whereas CD195 expression increased approximately 6-fold in AMs but not IMs (Figure 5F). As CD107a expression remains unchanged during BCG infection, it may serve as a reliable surface marker for AMs with enhanced mycobacterial uptake capacity. In support of this hypothesis, we gated on AMs (LiveCD45^+^CD64^+^F4/80^+^CD11b^lo^SiglecF^hi^CD11c^hi^) and, based on CD107a expression, we identified two AM subsets: CD107a^+^ and CD107a^−^, each comprising ∼50% of AMs (Figure 5G). CD107a^+^ and CD107a^−^ displayed similar FSC and SSC profiles (data not shown). Notably, the CD107a^+^ subset showed significantly higher frequencies of BCG-SYTO24^+^ macrophages and elevated MFI for BCG uptake (Figures 5H–5J). Our results demonstrate that CD107a identifies a hyperphagocytic subset of AMs *in vivo* with enhanced BCG uptake. Following BCG exposure, CD107a^+^ AMs significantly upregulate surface CD195 expression, highlighting their role in mycobacterial interactions and suggesting CD107a and CD195 as key markers of hyperphagocytic macrophages in mycobacteria infection.

**Figure 5:**
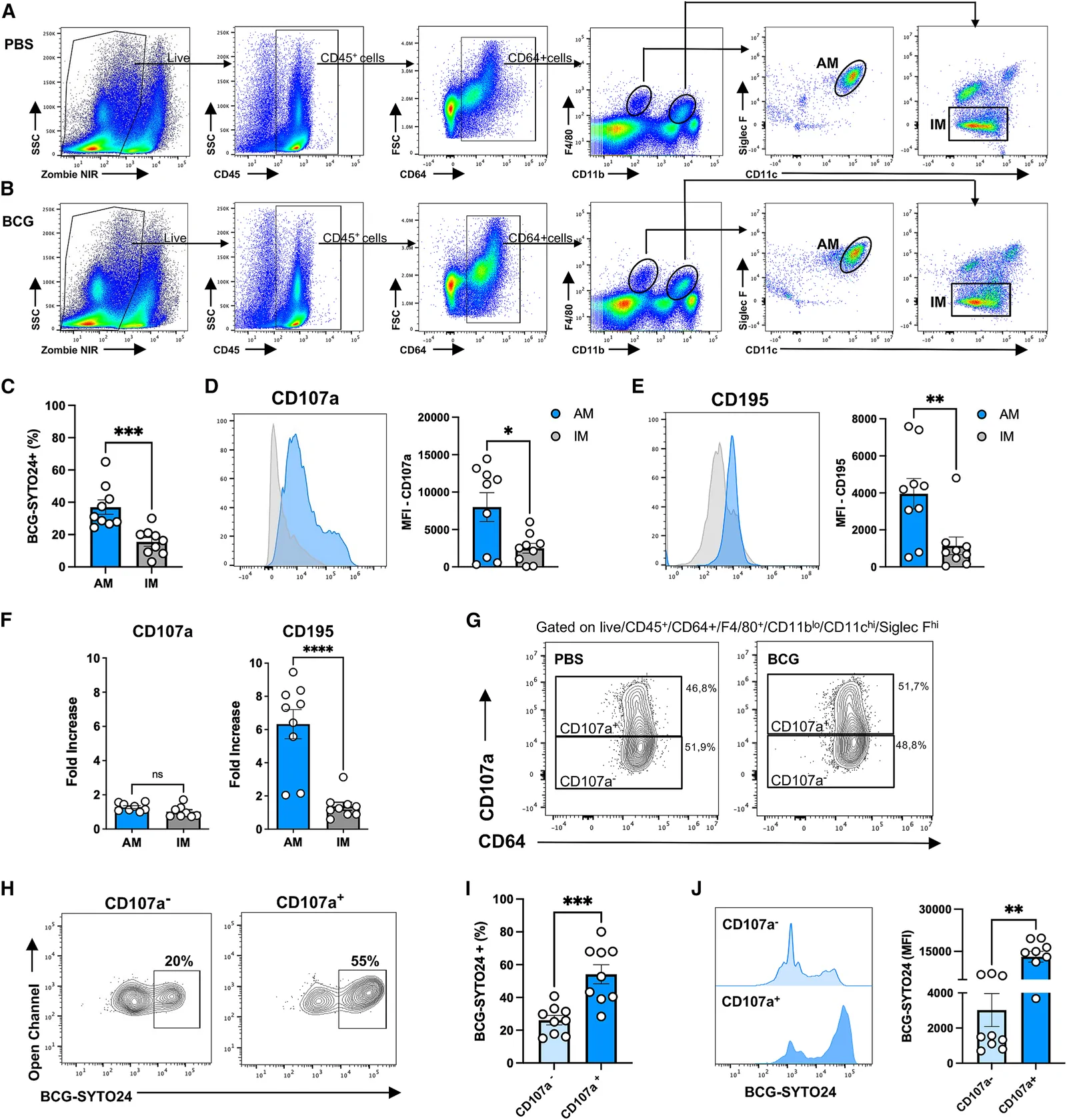
CD107a and CD195 expression in alveolar macrophages is associated with enhanced mycobacterial phagocytosis *in vivo* (A) Lung cell preparations from C57BL/6J mice exposed intratracheally (i.t.) to saline or *M*. *bovis* BCG (10^8^ CFU/mouse) were analyzed by flow cytometry. Gating strategy for identifying alveolar macrophages (AM; CD45^+^CD64^+^F4/80^+^CD11b^lo^SiglecF^hi^CD11c^hi^) and interstitial macrophages (IMs; CD45^+^CD64^+^F4/80^+^CD11b^hi^SiglecF^neg^CD11c^lo^) in total lung cell preparations from saline-exposed mice. (B) Gating strategy for identifying AM and IM populations in total lung cell preparations from BCG-exposed mice. (C) Frequency of BCG-SYTO24^+^AMs and IMs. (D) Representative histograms and MFI of CD107a expression in AMs and IMs from saline-exposed mice. (E) Representative histograms and MFI of CD195 expression in AMs and IMs from saline-exposed mice. (F) Fold change in surface receptor expression in AMs and IMs from BCG-infected mice relative to saline controls. (G) C57BL/6J mice were exposed intratracheally to saline or BCG for 18 h, and lung cells were analyzed by flow cytometry. Representative contour plots showing CD107a versus CD64 expression in AMs from saline- and BCG-exposed mice. (H) Representative contour plot showing BCG^+^cells within CD107a^−^ or CD107a^+^ AMs. (I) Frequency of BCG-SYTO24^+^cells in CD107a^−^ or CD107a^+^ AMs, as defined in H. (J) MFI of BCG-SYTO24 in CD107a^−^ and CD107a^+^ AMs at 18 h post-BCG exposure. Data in (A–J) were pooled from 4 independent experiments, with 9 mice per group. Statistical analyses were performed using Student’s *t* test. ∗∗*p* < 0.01; ∗∗∗*p* < 0.001; ∗∗∗∗*p* < 0.0001.

## Discussion

Macrophage heterogeneity prompts questions about subset-specific roles in pathogen interactions. We identified a hyperphagocytic FSC^lo^SSC^lo^(F4/80^lo^CD11b^lo^) BMM subset with elevated CD107a and CD195 expression, indicating enhanced mycobacterial uptake (Figures 2C–2E). *In vivo*, CD107a^+^ AMs in BCG-infected mice showed higher mycobacterial loads, mirroring *in vitro* findings. These results suggest that CD107a and upregulation of CD195 mark hyperphagocytic macrophages, with potential implications for tuberculosis (TB) host defense strategies.

Transcriptomic analysis of FSC^lo^SSC^lo^ BMMs reveals enriched phagocytosis, actin cytoskeleton, including coronin 1a and fibronectin 1, as well as pro-inflammatory cytokine pathways (Figures 1N and 3C).^27^^,^^28^ Our transcriptomic analysis revealing enriched phagocytosis, actin cytoskeleton, and innate sensing pathways in macrophage subsets was substantiated by functional assays, including TNF and IL-6 production in response to TLR2, TLR4, and TLR5 ligands (Figure 3B) and elevated cytokine secretion post-Mtb infection (Figure 3D), confirming the pro-inflammatory and phagocytic specialization of each subset. Additional studies are required to further characterize the specific roles of enriched genes linked to pathogen recognition and internalization, such as *Tlr2* and *Pfn1*, in driving the selective mycobacterial uptake by FSC^lo^SSC^lo^ macrophages. While both macrophage subsets exhibited high expression of phagocytic receptors, they demonstrated distinct transcriptional programs associated with phagocytosis, suggesting previously unknown entry mechanisms utilized by mycobacteria.

While virulent *M. tuberculosis* strains (e.g., H37Rv) exhibit enhanced immune evasion compared to BCG (e.g., via ESX-1-mediated phagosomal perforation), core mechanisms of initial macrophage uptake, including actin-mediated phagocytosis and primary engagement of complement receptors, are conserved across attenuated (BCG, H37Ra) and virulent strains (H37Rv, Erdman). For example, studies have shown that both virulent and attenuated strains utilize complement receptors as the dominant pathway for entry, with virulent strains additionally recruiting mannose receptors in some contexts.^10^ *In vivo*, BCG infection reliably models AM uptake and recruitment of phagocytic subsets, mirroring key early events in TB pathogenesis.

The superior mycobacterial uptake by the FSC^lo^SSC^lo^ subset is closely associated with its enhanced binding capacity, indicating that stronger initial surface association may actively promote phagocytosis in this population. This is consistent with known mechanisms of mycobacterial entry, where stable receptor-mediated adhesion (e.g., via complement and mannose receptors) is a prerequisite for efficient actin polymerization and phagocytic cup closure. While flow-based assays cannot fully decouple binding from internalization without orthogonal controls (e.g., confocal z stack analysis), our data support a model in which enhanced adhesion in the FSC^lo^SSC^lo^ subset drives its mycobacteria-selective hyperphagocytosis.

Although the FSC^lo^SSC^lo^ subset represents only ∼20% of BMMs, it accounts for the majority of mycobacterial internalization. This phenotype is partially reduced by cytochalasin D, suggesting that actin polymerization is central to the hyperphagocytic behavior of FSC^lo^SSC^lo^ BMMs (Figures 2J–2M). This partial sensitivity aligns with the complex, multireceptor nature of mycobacterial phagocytosis, which can involve partial actin-independent components, differing from stronger inhibition reported for Gram-positive cocci^29^^,^^30^ or zymosan (this study). Their heightened sensitivity to actin disruption (Figures 2K–2L) may relate to the enriched expression of actin cytoskeleton regulators such as *Pfn1* and *Coro1a* (Figure 1N), suggesting distinct actin dynamics in this subset. Consistent with a broader role for actin polymerization, cytochalasin D also reduced phagocytosis of fluorescently labeled zymosan or *Salmonella* particles. However, FSC^lo^SSC^lo^ macrophages displayed comparatively lower uptake of these particles than FSC^hi^SSC^hi^ cells (Figures 2D and 2E), suggesting that their dominance in mycobacterial uptake is unlikely to reflect a generalized increase in phagocytic capacity. We speculate that upstream recognition or targeting mechanisms may contribute to the selective engagement of mycobacteria by this subset.

Transcriptomic enrichment for GO terms related to “phagocytosis” and “actin cytoskeleton reorganization” in the FSC^lo^SSC^lo^ subset (Figures 1M and 1N) therefore reflects a distinct functional program rather than indiscriminate hyperphagocytosis. Functional assays confirmed preferential uptake of mycobacteria (Mtb/BCG) by FSC^lo^SSC^lo^ cells, whereas *Salmonella* and zymosan were more efficiently internalized by FSC^hi^SSC^hi^ macrophages, which express higher levels of complementary receptors such as *Marco*, *Msr1*, and *Cd36* (Figure 1N). These patterns suggest subset-specific phagocytic specialization: FSC^lo^SSC^lo^ macrophages appear optimized for actin-mediated handling of mycobacteria, potentially involving Arp2/3-associated mechanisms and C-type lectins, while FSC^hi^SSC^hi^ cells preferentially engage pathways suited for other microbial particles. Notably, the transcriptomic enrichment of actin regulators in FSC^lo^SSC^lo^ macrophages is correlative, and direct functional studies will be required to establish their specific roles in driving this phenotype. Elevated *Tlr2* expression in FSC^lo^SSC^lo^ macrophages fuels robust TNF and IL-6 with low IL-10 production, suggesting a pro-inflammatory environment that mycobacteria might exploit.^31^^,^^32^^,^^33^ The earlier peak of TNF and IL-6 production by FSC^lo^SSC^lo^ macrophages at 24 h post-Mtb infection, compared to the delayed IL-6 peak at 48 h in FSC^hi^SSC^hi^ macrophages (Figure 3D), suggests a rapid pro-inflammatory response that may enhance early mycobacterial control in TB, though sustained inflammation could contribute to tissue pathology if unchecked. Yet, their IFN-γ-induced mycobactericidal activity matches FSC^hi^SSC^hi^ cells (Figure 3G), indicating intact IFN-γ-mediated effector functions. *In vivo*, rapid BCG binding by CD107a^+^ AMs as early as 2 h post-infection (data not shown) suggests a pre-primed hyperphagocytic state, potentially amplifying early mycobacterial interactions in the lung.

While high surface CD107a expression strongly correlates with and marks the hyperphagocytic FSC^lo^SSC^lo^ subset (Spearman’s ρ = 1.0, *p* = 0.0028; Figures 4E and 4F), our data do not demonstrate a direct causal role for CD107a in mediating uptake. Instead, it likely reflects heightened phagosomal activity, lysosomal biogenesis, or membrane dynamics associated with superior actin-driven phagocytosis in this population. Future conditional knockouts or CRISPR screens will clarify this receptor’s role. Moreover, CD195-deficient BMMs showed no defect in mycobacterial uptake (Figures 4H and 4I). This result indicates that CD195 primarily serves as a phenotypic marker of this specialized population rather than a direct functional driver or receptor mediating uptake. The specific receptor(s) responsible for the observed hyperphagocytosis remain to be identified and may involve other differentially expressed molecules (e.g., C-type lectins such as Mincle) or synergistic receptor engagement. Given the role of CD195 in cell migration, it is possible that CD107a^+^CD195^hi^ AMs may act as “Trojan horses,” facilitating mycobacterial dissemination. For instance, Kirby et al.^34^ showed that a subset of AM migrates to draining lymph nodes in a *Streptococcus pneumoniae* lung infection model. Future studies on macrophage trafficking and mechanistic roles of CD107a and CD195 are needed to further elucidate their contributions to mycobacterial interactions.

Our findings rely on mouse models, which may not fully recapitulate human macrophage biology, and short-term infection protocols that do not reflect the chronic nature of TB. Furthermore, although virulent *M. tuberculosis* H37Rv was used in key *in vitro* phagocytosis experiments (Figure 2), biosafety constraints required the use of attenuated *M. bovis* BCG for *in vivo* validation and several functional assays.

The *in vivo* experiments were performed at 18 h post-exposure to specifically examine early phagocytic events. While this acute model highlights the enhanced phagocytic capacity of resident CD107a^+^ AMs during initial uptake, it remains to be determined whether recruited monocyte-derived IMs^35^^,^^36^^,^^37^^,^^38^ can acquire a similar hyperphagocytic phenotype during the chronic phases of TB infection. Nevertheless, our findings define a previously unrecognized hyperphagocytic macrophage subset and establish CD107a/LAMP1 as a reliable surface marker, providing mechanistic insights into mycobacterial uptake and potential targets for intervention.

### Limitations of the study

The present study does not establish a direct functional role for CD107a in mediating mycobacterial uptake. While transcriptomic analyses identified candidate pathways associated with the hyperphagocytic phenotype, the individual contribution of these molecules to selective mycobacterial uptake remains to be determined. Our experiments primarily focused on the early stages of macrophage-mycobacteria interactions using murine BMMs, so we may have missed human-specific interaction pathways.

## Resource availability

### Lead contact

Requests for further information and resources should be directed to and will be fulfilled by the lead contact, André Luiz Barbosa Báfica (andre.bafica@ufsc.br).

### Materials availability

This study did not generate new unique reagents.

### Data and code availability

• RNA-seq data are deposited at the Gene Expression Omnibus repository under accession # PRJNA1294819.
• This paper does not report original code.
• Any additional information required to reanalyze the data reported in this paper is available from the lead contact upon request.

## Acknowledgments

We express our gratitude to Prof. Juliano Ferreira (UFSC) for providing animal housing facilities, Prof. Dario Zamboni (FMRP-USP) for supplying BMs from CD195-deficient mice, and Prof. Daniel Mucida (Rockefeller University) for granting access to RNA sequencing facilities. We sincerely thank Dr. Alan Sher (National Institutes of Health) for his insightful and constructive comments. This work was funded by Howard Hughes Medical Institute – Early Career Scientist (A.L.B.B.; 55007412) and CNPq/PQ Scholars (A.L.B.B., D.S.M., and E.L.d.R.).

## Author contributions

C.E., H.M.S., and A.L.B.B. designed the research; C.E., G.L., H.M.S., E.L.M., D.A.G.B.M., B.K.B., L.Z.M., M.C.C.C., B.S.R., M.S., G.S., and J.B. performed the research; Y.K.T.d.M., D.S.M., and E.L.d.R. contributed new reagents/analytic tools; C.E., G.L., H.M.S., M.S., E.L.d.R., and A.L.B.B. analyzed the data; and C.E. and A.L.B.B. wrote the paper.

## Declaration of interests

The authors declare no competing interests, with their salaries or scholarships funded by their universities, and they also pursue private and government grants.

## STAR★Methods

### Key resources table

**Table:** 

REAGENT or RESOURCE	SOURCE	IDENTIFIER
**Antibodies**		

Anti-CD11b, clone M1/70, PE	BD Biosciences	Cat# 557397
Anti-CD11c, clone HL3, PE	BD Biosciences	Cat# 557401
Anti-CD11c, clone HL3, BV480	BD Biosciences	Cat# 565627
Anti-CD40, clone 3/23, PE	BD Biosciences	Cat# 553791
Anti-CD44, clone IM7, APC	BD Biosciences	Cat# 553991
Anti-CD45, clone 30-F11, APC	BD Biosciences	Cat# 559864
Anti-CD45, clone 30-F11, Per CP	BD Biosciences	Cat# 557235
Anti-I-A/I-E, clone 2G9, FITC	BD Biosciences	Cat# 553623
Anti-Ly6G, clone 1A8, PerCP-Cy5.5	BD Biosciences	Cat# 560602
Anti-CD107a, clone 1D4B, BV421	BD Biosciences	Cat# 564347
Anti-CD107a, clone 1D4B, FITC	BD Biosciences	Cat# 553793
Anti-CD107b, clone M3/84, FITC	BD Biosciences	Cat# 562061
Anti-TNF, clone MP6-XT22, PE	BD Biosciences	Cat# 554419
Anti-TREM-1, clone 174031, BV421	BD Biosciences	Cat# 747899
Fixable Viability Stain 660	BD Biosciences	Cat# 564405
Anti-CD115, clone AFS98, Per CP 710	Thermo Fisher	Cat# 46115282
Anti-CD11b, clone M1/70, BV421	BioLegend	Cat# 101251
Anti-CD11c, clone N418, Per CP	BioLegend	Cat# 117325
Anti-CD64, clone X54-5/7.1, FITC	BioLegend	Cat# 139316
Anti-CD68, clone FA-11, PE	BioLegend	Cat# 137013
Anti-CD69, clone H1-2F3, PerCP-Cy5.5	BioLegend	Cat# 104522
Anti-CD74, ln1/CD74 Alexa Fluor 488	BioLegend	Cat# 151005
Anti-CD88, clone 20/70, PE-Cy7	BioLegend	Cat# 135810
Anti-CD107a, clone 1D4B, PE-Cy7	BioLegend	Cat# 121619
Anti-CD138, clone 281-2, APC	BioLegend	Cat# 142506
Anti-CD163, clone S15049, PE	BioLegend	Cat# 155307
Anti-CD170, clone S17007L, APC-Cy7	BioLegend	Cat# 155531
Anti-CD182 (CXCR2), clone TG11/CXCR2	BioLegend	Cat# 129103
Anti-CD195, clone HM-CCR5, APC	BioLegend	Cat# 107012
Anti-CD195, clone HM-CCR5, PE	BioLegend	Cat# 107006
Anti-CD206, clone C068C2, FITC	BioLegend	Cat# 141704
Anti-CD207, clone 4C7, PE-Cy7	BioLegend	Cat# 144209
Anti-F4/80, clone BM8, BV421	BioLegend	Cat# 123132
Anti-Ly6C, clone HK1.4, Alexa Fluor 488	BioLegend	Cat# 128021
Zombie NIR	BioLegend	Cat# 423105

**Bacterial and virus strains**		

*Mycobacterium tuberculosis* - H37Rv	Báfica Lab stock	–
*Mycobacterium bovis* BCG – Danish strain	Báfica Lab stock	–
*Salmonella enterica* serovar Typhimurium	ATCC	14028

**Chemicals, peptides, and recombinant proteins**		

Murine- colony stimulation factor (M-CSF)	PeproTech	31502
Murine interferon gamma (IFN-γ)	BD Pharmigen	554587
FITC-conjugated Zymozan	Invitrogen	Z2841
SYTO 24	Thermo Fischer	S7559
Cytochalasin D	Sigma-Aldrich	C8273
Cytochalasin D	Invitrogen	PHZ1063
Pam3Cys-SK4	EMC Microcollections	L2000
Ultra-pure LPS (*E. coli* 0111:B4)	Invivogen	tlrl-eblps
Flagellin (from *S. typhimurium*)	Invivogen	tlrl-epstfla
Liberase CI	Roche	135476000
Brefeldin A (GolgiPlug™)	BD Bioscience	555029
Accutase	Sigma-Aldrich	6964
Trizol	Invitrogen	10296010
DNAse I	Qiagen	79254
Phosphate Saline Buffer	Gibco	70011044
ProLong Gold antifade reagent containing DAPI	Invitrogen	P36935
DMEM high glucose	Gibco	11965092
Penicillin/streptomycin	Gibco	15070063
HEPES	Gibco	15630080
Fetal Bovine Serum (FBS)	Gibco	12657029
L-glutamine	Gibco	A2916801
Sodium pyruvate	Gibco	11360070

**Critical commercial assays**		

RNeasy Plus Micro kit	Qiagen	74034
ELISA TNF	Thermo Fischer	88-7324-88
ELISA IL-6	Thermo Fischer	88-7064-88
ELISA IL-10	Thermo Fischer	88-7105-88

**Deposited data**		

RNA sequencing data (raw and analyzed data)	This paper	GEO: # PRJNA1294819

**Experimental models: Cell lines**		

L-929	BCRJ- Cell Bank of Rio de Janeiro	N/A
Mouse C57BL/6J	CEMIB/ UNICAMP – Laboratory Animals Research Center	N/A
Graph Pad Prism 9.5.0	Graph Pad	https://www.graphpad.com/
Flow Jo 10.8.1	Becton Dickinson &Company	https://www.flowjo.com/
ImageJ	National Institute of Health	https://imagej.nih.gov/ij/

### Experimental model and study participant details

All animal-related protocols were approved by the Committee of Ethics on the Use of Animals from Federal University of Santa Catarina (CEUA/UFSC/#9566210722). Female and male C57BL/6J mice, 8–12 weeks of age and weighing 20–32 g, were housed under specific pathogen-free conditions at the Animal Facility of the Department of Pharmacology, Federal University of Santa Catarina. Mice were maintained under a 12-h light/12-h dark cycle at 22°C ± 2°C with *ad libitum* access to food and water. Each cage housed a maximum of five animals, and all cages were provided with environmental enrichment. Trained staff conducted daily welfare checks, enabling prompt identification of any signs of illness and ensuring animals were treated humanely at all times.

### Method details

#### Preparation of bacteria

Frozen stocks of *Mycobacterium tuberculosis* (Mtb, H37Rv strain), *M. bovis* BCG (BCG, Danish strain), and *Salmonella enterica* (ATCC 14028) were maintained in biosafety containment facilities at Federal University of Santa Catarina. Briefly, mycobacterial cultures were grown in Middlebrook 7H9 liquid medium supplemented with OADC for 15-21 days at 37°C until reaching ideal optical density. Bacteria concentration was determined by plating onto 7H10 Middlebrook agar. *S. enterica* cultures were grown in BHI liquid medium, and bacteria concentration was determined by plating onto LB agar. For fluorescence assays, bacterial suspensions were incubated with SYTO24 (1μM) for 1 hour at 37°C, as previously described,^39^ and then washed with sterile PBS (Gibco, 70011044) prior to infections. SYTO24 labeling, used for fluorescence-based detection of *M. tuberculosis*, *M. bovis* BCG, and *S. enterica*, does not alter bacterial infectivity or growth, as previously validated.^39^^,^^40^ Heat-killed BCG was obtained by boiling bacterial suspension at 95°C for 10 minutes.

#### Preparation of L929 cell conditioned medium

L929 murine cells were cultured in DMEM (Gibco, 11965092) supplemented with 10% heat-inactivated Fetal Bovine Serum (FBS, Gibco, 12657029), 2 mM L-glutamine (Gibco, A2916801), 1 mM sodium pyruvate (Gibco, 11360070), and 1.2 mM HEPES (Gibco, 15630080) for three passages following thawing from cryogenic storage. A total of 3 × 10^6^ cells were seeded in a 150 cm^2^ culture flask containing 50 mL of complete DMEM. After seven days, the medium was replaced with an additional 50 mL of fresh DMEM and maintained for another seven days. The resulting L929-conditioned medium was then filtered using a 0.22 μm filter, aliquoted into 50 mL Falcon tubes, and stored at −20°C until use.

#### Generation of bone marrow-derived macrophages

Bone marrow-derived macrophages (BMMs) were generated from 8- to 12-week-old male or female C57BL/6J mice by harvesting tibia and femur bones. Whole bone marrow (BM) cells were cultured in DMEM supplemented with 20% L929 cell-conditioned medium, 2 mM L-glutamine, 1 mM sodium pyruvate, 1.2 mM HEPES, 10% heat-inactivated FBS, and 1 U/mL penicillin/streptomycin (Gibco, 15070063). Cells were maintained in Petri dishes for seven days, with an additional 5 mL of supplemented medium added on Day 5. On Day 7, non-adherent cells were removed by washing with sterile PBS, and adherent macrophages were used for experiments. In a separate set of experiments, BMMs were generated by culturing whole bone marrow cells in the presence of recombinant murine M-CSF (30 ng/mL) (PeproTech, 31502).

#### Antibodies and fluorescent conjugates

The following antibodies were purchased from BD Biosciences: anti- CD11b (clone M1/70) PE, 557397; anti-CD11c (clone HL3) PE, 557401, BV480, 565627 ; anti-CD40 (clone 3/23) PE, 553791; anti-CD44 (clone IM7) APC, 553991; anti-CD45 (clone 30-F11) APC and PercP, 559864, 557235 ; anti-I-A/I-E (clone 2G9) FITC, 553623; anti-Ly6G (clone 1A8) PerCPp-Cy5.5, 560602; anti-CD107a (clone 1D4B) BV421 and FITC, 564347, 553793 ; anti-CD107b (clone M3/84) FITC, 562061; anti-TNF (clone MP6-XT22) PE, 554419; anti-TREM-1(clone 174031) BV421, 747899 and Fixable Viability Staining 660, 564405. The following antibody was purchased from Thermo Fisher: anti-CD115 (clone AFS98) PercP 710, 46115282; The following antibodies were purchased from BioLegend: anti-CD11b (clone M1/70) BV421, 101251; anti-CD11c (clone N418) PerCP, 117325; anti-CD64 (clone X54-5/7.1) FITC, 139316; anti-CD68 (clone FA-11) PE, 137013; anti-CD69 (clone H1-2F3) PerCP Cy5.5, 104522; anti-CD74 (clone) Alexa Fluor 488, 151005; anti-CD88 (clone 20/70) PECy7, 135810; anti-CD107a (clone 1D4B) PECy7, 121619; anti-CD138 (clone 281-2) APC, 142506; anti-CD163 (clone S15049) PE,155307; anti-CD170 (clone S17007L) APC-Cy7,155531; anti-CD182 (clone TG11/CXCR2), 129103, anti-CD195 (HM-CCR5) APC and PE, 107012, 107006; anti-CD206 (clone C068C2) FITC, 141704; anti-CD207 (clone 4C7) PE-Cy7, 144209; anti-F4/80 (clone BM8) BV421, 123132; anti-Ly6C (clone HK1.4) Alexa fluor 488; 128021; and Zombie NIR, 423105).

#### Flow cytometry and cell sorting and gating strategy

BMMs were detached from plates using accutase (Sigma-Aldrich, 6964), washed once with PBS, and resuspended in FACS buffer (PBS + 1% FBS). Samples were pre-incubated with Fc block (BD Bioscience, 552141) for 10 minutes on ice to minimize nonspecific binding. FACS sorting was performed using a Melody or ARIA II sorter with a 100-μm nozzle (Becton Dickinson). The gating strategy included exclusion of dead cells using Zombie NIR staining, selection of leukocytes as CD45^+^ cells, and subsequent gating based on forward scatter (FSC) versus side scatter (SSC) parameters. Within the final gates, the median fluorescence intensity (MFI) of F4/80 and CD11b was measured. Flow cytometry data were acquired using a FACSVerse (Becton Dickinson) or a Cytek® Northern Lights flow cytometer, and data analysis was performed with FlowJo software (version 10.8.1).

#### RNA isolation and sequencing

Total RNA of sorted SSC^hi^FSC^hi^F4/80^hi^CD11b^hi^ and SSC^lo^FSC^lo^F4/80^lo^CD11b^lo^ macrophages, derived from three independent cell cultures, was extracted using TRIzol LS (Invitrogen; 10296010) treated with DNase I (Qiagen; 79254), and purified with the RNeasy Plus Micro kit (Qiagen; 74034). RNA quality was assessed using Pico Bioanalyser chips (Agilent, 5067-1513). Only RNAs displaying integrity numbers of 9 or higher were used for downstream procedures. RNAseq libraries were prepared using the Nugen Ovation Trio low input RNA Library Systems V2 (Nugen, 0507-08) according to the manufacturer’s instructions by the RU Genome Technology Center. Pooled libraries were sequenced as 50-nucleotide, paired-end reads on an Illumina HiSeq 2500 using v4 chemistry.

#### RNAseq data quality assessment and visualization

Illumina sequencing adapters and reads with Phred quality scores <20 were removed with Trimmomatic. Trimmed reads were mapped to the *Mus musculus* genome Ensembl annotation release 91 using STAR v2.5.3a with default settings. The number of reads uniquely mapping to each gene feature in the corresponding annotation file was determined using featureCounts. The resulting count tables were passed to R for further analyses. RNAseq data are deposited at the Gene Expression Omnibus repository under accession #PRJNA1294819.

#### Differential expression and gene ontology analyses

Consistency between replicates was checked using PCA and Euclidean distance–based hierarchical clustering on normalized counts. Weakly expressed genes, defined as having < 3 fragment per kilobase of transcript per million mapped reads in at least one group of replicates, were removed from differential expression analysis. Differential expression analyses were performed using the DESeq2 package. Genes were considered significant when the adjusted *p*-value was < 0.05. The set of significant genes for each contrast of interest was passed to enrichR and ClusterProfiler for functional gene enrichment analyses and heatmaps were performed using Pheatmap.

#### Phagocytosis experiments

For flow cytometry-based quantification of mycobacterial phagocytosis by BMMs, cells were infected with SYTO24-stained Mtb or BCG at different multiplicity of infection (MOI). Unlabeled Mtb or BCG were used as controls for flow cytometry experiments. At different time points post-infection, cells were extensively washed with cold PBS and stained with antibody panels for flow cytometry analysis. In a separate set of experiments, BMMs were incubated with 50 μg/ml FITC-conjugated zymosan (Invitrogen, Z2841) for 4 hours or *Salmonella*-SYTO24 (MOI 10) for 30 min at 37°C, followed by extensive PBS washing before flow cytometry analysis. In a subset of experiments, cytochalasin D (Sigma-Aldrich, C8273 for Figure 2 and Invitrogen, PHZ1063 for Figure S1B) was added 30 minutes prior to exposure to SYTO24-stained *M. bovis* BCG, FITC-conjugated zymosan or *Salmonella*-SYTO24. Cells were then incubated with bacteria for 4 hours at 37°C, followed by extensive PBS washing before flow cytometry analysis. To quantify mycobacterial uptake, MFI and frequency of positive macrophages were obtained by gating on specific cell subpopulations. Gating strategy was performed using controls of macrophage exposed to unlabeled particles. For microscopy-based quantification of mycobacterial uptake, BMMs were isolated from WT mice and allowed to adhere overnight onto poly-L-lysine-coated glass coverslips (Molecular Probes). Cells were then infected with SYTO24-stained Mtb at a MOI of 10 for 4 hours, followed by extensive wash and fixation with ProLong Gold antifade reagent containing DAPI (Invitrogen, P36935). The number of Mtb-infected cells was quantified across 50 fields (averaging 2 bacilli per cell) using a 100× objective on a BX41 microscope (Olympus Life Science). Data were expressed as the cell area versus the number of bacilli/cells.

#### Cytokine production measurements

BMMs were seeded in 96-well flat-bottom plates at a density of 1×10^6^ cells/mL and infected with Mtb at a MOI of 3 for 4 hours. Following extensive washing to remove extracellular bacteria, cells were cultured for different time points. TNF, IL-6 and IL-10 were quantified in culture supernatants by commercially available ELISA. In separate experiments, BMMs were stimulated with Pam3Cys (S-[2,3-bis(palmitoyloxy)-(2-RS)-propyl]-*N*-palmitoyl-(R)-Cys-(S)-Ser-Lys4-OH, trihydrochloride) (100 ng/mL, EMC Microcollections), ultra-pure LPS (*E. coli* 0111:B4, Invivogen, tlrl-3pelps) (100 ng/mL), flagellin (100 ng/mL, Invivogen, tlrl-epstfla-5), or heat-killed BCG (MOI 10) for 6 hours at 37°C, with brefeldin A (BD GolgiPlug™, 555029) added during the final 4 hours. For intracellular TNF staining, single-cell suspensions were surface-stained, fixed, and permeabilized. Intracellular TNF was detected using anti-TNF monoclonal antibody (clone MP6-XT22). The percentage of TNF^+^ macrophages was determined by gating on specific cell subpopulations.

#### Mtb killing assays

BMMs were seeded in 96-well flat-bottom plates at a density of 1×10^6^ cells/mL and infected with Mtb at a MOI of 3 for 4 hours. Following extensive washing to remove extracellular bacteria, infected macrophages were treated with recombinant murine IFN-γ (BD Pharmingen, 554587). At 24 hours and 48 hours post-infection, cells were washed and lysed with 1% saponin. Bacterial burden was assessed by plating lysates onto 7H10 Middlebrook agar for CFU enumeration. Nitrite production was measured using the Griess reaction,^41^ with absorbance recorded at 540 nm.

#### *In vivo* infection model and flow cytometry experiments

Female and male C57BL/6J mice (8–12 weeks old) were housed under specific pathogen-free conditions at the animal facility of the Pharmacology Department, Universidade Federal de Santa Catarina. For infection, *M. bovis* BCG was suspended in PBS, and bacterial clumps were dissociated using a sterile needle attached to a syringe by repeatedly passing the suspension through the needle. Mice were infected via the intratracheal (i.t.) route with a total of 10^8^ CFU in PBS (50 μL) using a 0.25 mm needle gauge. At 18 h post-infection, whole lungs were collected and enzymatically digested with Liberase CI (Roche, 135476000) for 20 minutes at 37°C. Single-cell suspensions were prepared by passing the tissue through 100 μm cell strainers, and 2×10^6^ cells were used for flow cytometry staining. Gating strategy: Dead cells were excluded using Zombie NIR viability staining, and leukocytes were identified by gating on CD45^+^ cells. Within the CD45^+^ population, F4/80^+^CD11b^lo^ and F4/80^+^CD11b^hi^ macrophage subsets were defined. Alveolar macrophages (AMs) were identified as Siglec-F^hi^ cells within the F4/80^+^CD11b^lo^ gate. AM subsets were further characterized by CD107a and CD195 expression, delineating AM1 and AM2 populations.

### Quantification and statistical analysis

Statistical analysis was performed using GraphPad Prism software (version 10). The Shapiro-Wilk test was used to assess normality of the data. For comparisons between two groups, unpaired or paired Student’s t-tests were applied when data were normally distributed; when normality was not met (Shapiro-Wilk, *p* < 0.05), the Mann-Whitney U test (unpaired) or Wilcoxon matched-pairs signed rank test (paired) was used. For comparisons involving more than two groups or conditions, one-way ANOVA or two-way ANOVA was performed, followed by Tukey’s post hoc test when data passed normality. Statistical significance was defined as *p* < 0.05 (two-tailed). To evaluate the magnitude and biological relevance of the findings, Cohen’s d was calculated as a standardized effect size, and 95% confidence intervals were computed for median differences and fold changes where applicable. The exact sample size (n), and statistical significance for each experiment are reported in the corresponding figure legends.
