# Supplementary material for: A specialized CD107a+ macrophage subset drives hyperphagocytosis of mycobacteria
Source: iScience. 2026 Sep 25;29(10):117622. doi: 10.1016/j.isci.2026.117622 (PMC13663896; doi:10.1016/j.isci.2026.117622)
Supplement: Supplementary file 1 [file mmc1.pdf]

iScience, Volume *29*

Supplemental information

A specialized CD107a$^+$ macrophage subset drives hyperphagocytosis of mycobacteria

Carolina Eto, Gabriela Luiz, Hernandez M. Silva, Eduarda L. Munari, Daniel A.G.B. Mendes, Bianca K. Beck, Lucas Z. Mascarin, Marick Starick, Maria Cecília C. Canesso, Bernardo S. Reis, Guilherme Silveira, Juliano Bordignon, Yonne K.T. de Menezes, Daniel S. Mansur, Edroaldo Lummertz da Rocha, and André Luiz Barbosa Báfica

Supplementary Figure 1. Eto et al.

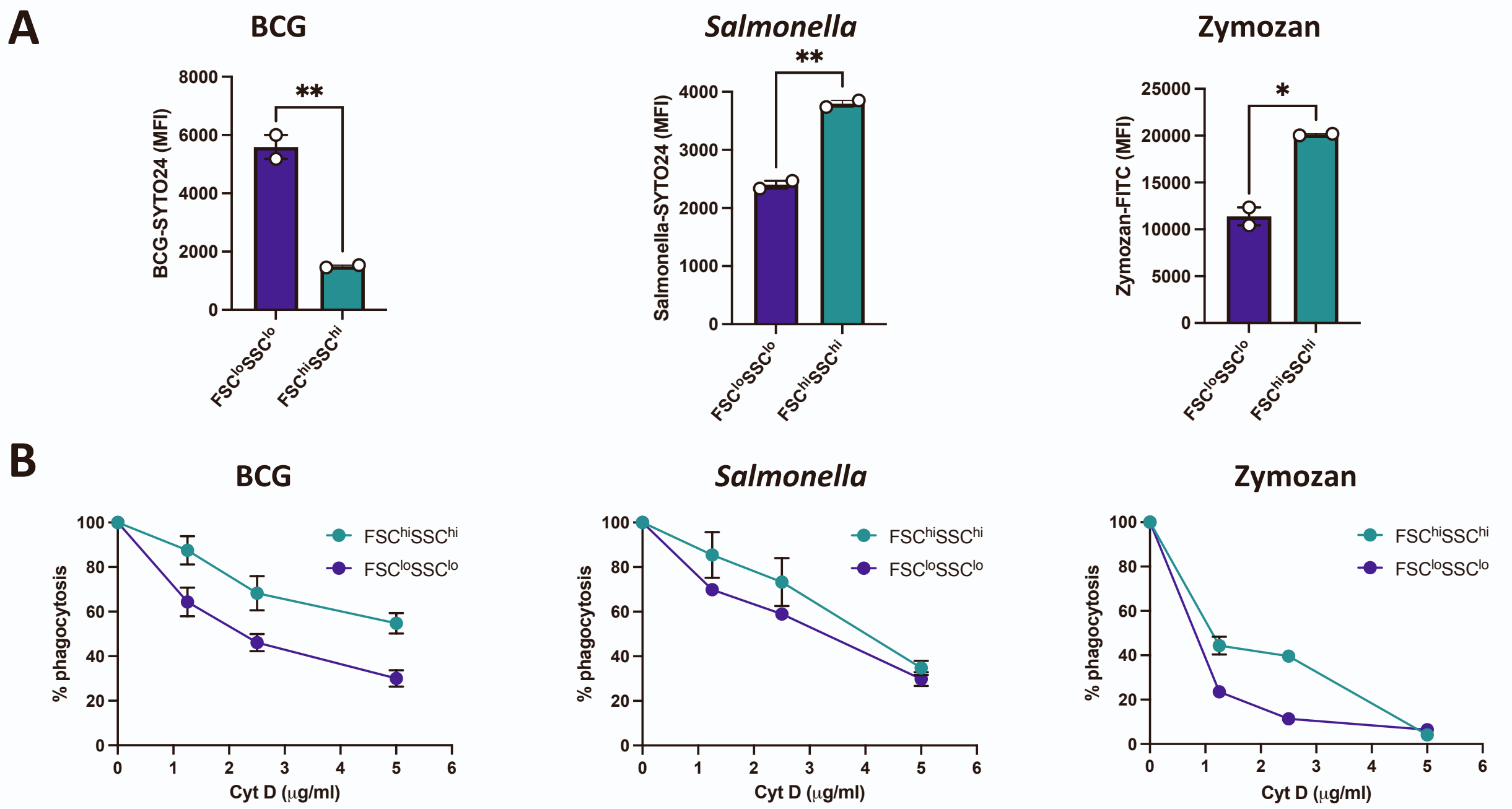

Supplementary Figure 1. Cytochalasin (Cyt D) inhibits phagocytosis of BCG, Salmonella and zymosan by $FSC^{hi}SSC^{hi}$ and $FSC^{lo}SSC^{lo}$ macrophages. Bone marrow–derived macrophages (BMMs) were pretreated with cytochalasin D (1, 2.5, or 5 μg/mL) for 30 min prior to exposure to BCG-SYTO24 (MOI 10, 4 h), *Salmonella*-SYTO24 (MOI 10, 30 min), or zymosan-FITC (50 μg/mL, 4 h), followed by flow cytometry analysis. **(A)** Mean fluorescence intensity (MFI) of BCG, *Salmonella*, or zymosan uptake in $FSC^{hi}SSC^{hi}$ and $FSC^{lo}SSC^{lo}$ macrophages. **(B)** Dose–response effect of cytochalasin D (1, 2.5, or 5 μg/mL) on phagocytosis by $FSC^{hi}SSC^{hi}$ and $FSC^{lo}SSC^{lo}$ macrophages. Data represent mean ± SEM from two independent mice.
